# Biopolymer-Inspired N-Doped Nanocarbon Using Carbonized Polydopamine: A High-Performance Electrocatalyst for Hydrogen-Evolution Reaction

**DOI:** 10.3390/polym12040912

**Published:** 2020-04-15

**Authors:** Duong Nguyen Nguyen, Uk Sim, Jung Kyu Kim

**Affiliations:** 1 School of Chemical Engineering, Sungkyunkwan University (SKKU), Suwon 16419, Korea; nguyendun@gmail.com; 2 Department of Materials Science & Engineering, Engineering Research Center and Optoelectronics Convergence Research Center, College of Engineering and College of AI Convergence, Chonnam National University, Gwangju 61186, Korea

**Keywords:** polydopamine, PDA, carbon nanosheet, nitrogen-doped carbon, electrocatalysts, hydrogen-evolution reaction

## Abstract

Hydrogen-evolution reaction (HER) is a promising technology for renewable energy conversion and storage. Electrochemical HER can provide a cost-effective method for the clean production of hydrogen. In this study, a biomimetic eco-friendly approach to fabricate nitrogen-doped carbon nanosheets, exhibiting a high HER performance, and using a carbonized polydopamine (C-PDA), is described. As a biopolymer, polydopamine (PDA) exhibits high biocompatibility and can be easily obtained by an environmentally benign green synthesis with dopamine. Inspired by the polymerization of dopamine, we have devised the facile synthesis of nitrogen-doped nanocarbons using a carbonized polydopamine for the HER in acidic media. The N-doped nanocarbons exhibit excellent performance for H_2_ generation. The required overpotential at 5 mA/cm^2^ is 130 mV, and the Tafel slope is 45 mV/decade. Experimental characterizations confirm that the excellent performance of the N-doped nanocarbons can be attributed to the multisite nitrogen doping, while theoretical computations indicate the promotion effect of tertiary/aromatic nitrogen doping in enhancing the spin density of the doped samples and consequently in forming highly electroactive sites for HER applications.

## 1. Introduction

Hydrogen-evolution reaction (HER) has generated significant interest as a promising technology for efficient renewable energy conversion [1,2,3,4,5,6]. Currently, the most efficient catalysts for HER, in addition to Pt, are earth-abundant metals, such as transition-metal sulfides [7,8,9], carbides [10,11,12], phosphides [13,14,15], and borides [16,17,18], which can replace noble metals.

In recent years, nonmetallic heteroatom-doped carbon materials have been intensively studied for energy-related electrocatalytic water-splitting processes such as the oxygen-reduction reaction (ORR) [19,20,21], oxygen-evolution reaction (OER) [22,23,24], and HER [25,26,27] because of their excellent electrical conductivity, high surface area, tunable molecular structures, and strong tolerance to various electrolytes. More importantly, it is noteworthy that carbon materials can be engineered to exhibit specific catalytic capabilities for specific electrocatalytic reactions by varying the doping types, sites, and levels [21,22,23,27,28,29,30,31]. Although some of these have recently been developed as HER electrocatalysts [32,33,34], the employment of carbon-based nanomaterials as high-performance candidates has not been reported to date. Clearly, the HER performance of carbon materials is not comparable to that of metal-based catalysts and does not presently conform to the benchmarks [31,35] established by metal-based catalysts. In addition, previously reported carbon-based electrocatalysts still exhibit several limitations during the synthesis process. For example, the synthesis of CVD-graphene-based catalysts is significantly expensive and requires multiple gas sources with particular pressure control and multiple transfer processes after the synthesis [34]. (Reduced) graphene oxide-based catalysts are a satisfactory alternative to CVD-graphene-based catalysts; however, their synthesis is time-consuming, toxic, and highly exothermal [36,37]. Furthermore, carbon sources exert a significant influence on the preparation process and the (electro)chemical properties of the resultant electrocatalyst. Among the potential candidates, polydopamine (PDA) has emerged as a new carbon precursor in recent years [28,38,39,40,41]. PDA is the self-polymerized product of dopamine, which can be synthesized under mild aqueous conditions simulating marine conditions. Due to the presence of catechol groups, the resultant PDA exhibits strong adhesion to bulk substrates or organic and inorganic materials. Therefore, the PDA does not only serve as the functional layer for many applications [42,43,44] but is also a promising carbon source for the preparation of carbon-based materials. So far, although the structure of the PDA is still under debate, carbonized PDA (C-PDA) has been utilized as nitrogen-doped graphite [40,42].

In this study, we demonstrate that through a variety of chemical doping processes—active sites for the HER—can be generated in the C-PDA. Further, these active sites can enhance and control catalytic activity. By the self-polymerization of dopamine through the dipping method, PDA can be produced universally on the substrate surface [45]. This self-polymerization is so mild that the simple immersion of a target substrate (e.g., Cu foil in this study) in an aqueous solution containing dopamine immediately results in the spontaneous deposition of a PDA film. Owing to the layer structure [40], the thickness of the film can be engineered to the nanometer scale using the immersion time [38]. The calcination of the PDA is then achieved in a tube furnace at elevated temperatures in a nitrogen atmosphere. Because of the complexity of the oxidative self-polymerization of dopamine, the chemical disorder model has been preferentially used in recent studies to describe PDA [39,46,47]. However, after calcination, the C-PDA has been proposed as nitrogen-rich carbon nanosheets [40,48] using the semiconductor model. In addition, the C-PDA film can be easily transferred to arbitrary substrates (e.g., Si wafer) by employing polymethylmethacrylate (PMMA), thus presenting a significant benefit over CVD-graphene. In addition, the combination of experimental characterizations and density functional theory (DFT) calculations jointly confirm the origin of this activity enhancement. The results confirm that the different sites of nitrogen doping are critical in the formation of catalytically active sites and enhancements in charge transfer. Considering N is the most important dopant in carbon materials for electrocatalysis, this promising synthetic method is expected to establish a robust foundation for the further exploration and development of nanostructural PDA-based carbon materials for broader applications [28,49].

## 2. Experimental 

### 2.1. Materials 

All the commercially available reagents and solvents were purchased from Sigma–Aldrich (Seoul, Korea)), Acros Organics (Seoul, Korea), or TCI chemicals (Tokyo, Japan), and were used as received without any further purification. The target substrate was purchased from commercial sources. 

### 2.2. Synthetic Procedure

**Figure 1:**
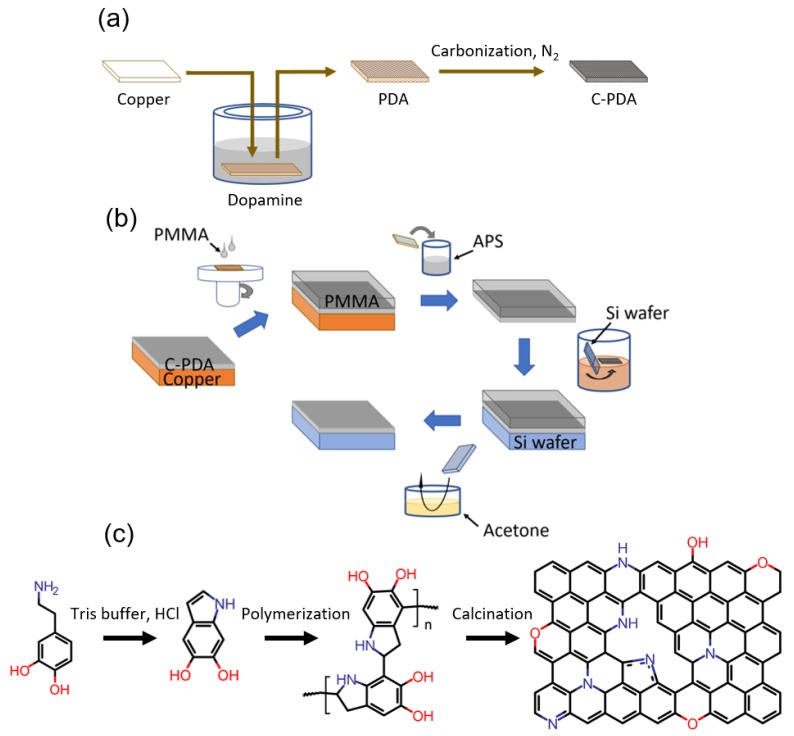
Fabrication of the carbonized polydopamine (C-PDA) for hydrogen generation. (**a**) Schematic of self-polymerization of dopamine on target substrate using the dipping method [38], (**b**) schematic showing the steps of the C-PDA film transfer onto Si wafer, (**c**) and the scheme of synthesis of N-doped carbon using dopamine as a precursor [50,51,52].

The PDA was synthesized according to the methods reported in previous literature [38], with some modifications (Figure 1a). In this study, a commercial copper foil was used as the substrate for coating the thin PDA film. After ultrasonic cleansing in acetone, isopropanol, and deionized (DI) water, the substrate was immersed in 45 ml of Tris buffer (tris (hydroxymethyl) aminomethane solution, 10 mM) containing 2 mg of dopamine chloride. The pH value of the Tris buffer was adjusted to 8.5 using HCl. The coating polymerization was conducted at 25 °C without mechanical stirring. After one hour, the substrates were removed from the solution, cleaned with DI water several times, and dried overnight at 70 °C in an oven. 

Substrates with PDA-coating were heat-treated under flowing nitrogen using a tube furnace. Using a typical heat treatment process, the system was heated at the heating rate of 10 °C/min, fixed at the 800 °C for an hour, and then cooled to room temperature naturally. 

### 2.3. Transfer of C-PDA onto Si Wafer

The C-PDA was fabricated onto the Si surface by the following procedure (Figure 1b). First, the PMMA was spin-coated on the C-PDA/copper substrate as a supporting layer. Then, the copper substrate was removed by immersing the PMMA/C-PDA/copper substrate in a diluted ammonium persulfate solution (0.2 M). After washing with DI water, the PMMA/C-PDA was then deposited on the Si wafer. Finally, the PMMA was removed by acetone, with the C-PDA as the remainder on the Si substrate. This sample was stored in a vacuum oven for further atomic force microscopy (AFM) characterization.

### 2.4. Material Characterization

The thin C-PDA film samples were collected on copper-coated carbon grids and examined using transmission electron microscopy (TEM) images obtained by using a JEM-2100 microscope (JEOL, Tokyo, Japan). AFM images were acquired under ambient conditions with a SPA-300HV model (Seiko, Tokyo, Japan) operated in the tapping mode. The Raman spectra were collected on a Renishaw micro-Raman spectroscope (Renishaw, Seoul, Korea) with a 514.5 nm Ar laser as the excitation source. The X-ray photoelectron spectroscopy (XPS) analysis was conducted on an ESCALAB 250 photoelectron microprobe (Thermo Scientific, Seoul, Korea) with monochromated Al Kα radiation (1486.6 eV). 

### 2.5. Electrochemical Measurement

Electrochemical measurements were achieved with a CHI 780 electrochemical analyzer (CH Instruments, Inc. Austin, TX, USA) using a standard three-electrode cell. A platinum foil and an Ag/AgCl (3 M NaCl) were used as the counter and reference electrodes, respectively. The working electrode was the glassy carbon (GC) tip (diameter 5.0 mm) of a rotating disk electrode (RDE). The RDE test was performed at 1000 rpm with a scan rate of 5 mV/s. For comparison, Pt/C was mixed under ultrasonication with a NAFION solution (5 wt.%, Alfa, Seoul, Korea) to form a well-dispersed catalyst “ink”. Then, 10 μl of the catalyst ink was drop-casted onto the polished GC tip. All the potentials were reported with respect to the reversible hydrogen electrode (RHE) in H_2_-saturated 1 M perchloric acid at 25 °C. 

### 2.6. Theoretical Calculation

The software packages ORCA 4.2.0 and Gaussian 09 were used for calculations [53]. The geometries of all initial models were optimized by the DFT method using the Perdew–Burke–Ernzerhof exchange-correlation functional. The Ahlrichs (def2-SVP) basis set was used for all atoms. The dispersion forces (the DFT-D3 method proposed by Grimme et al. [54]) were considered for dispersion correction. The resolution of identity (RI) approximation was used to boost the calculation; however, it should be noted that it produced an insignificant error. All calculations were achieved in a vacuum environment. 

## 3. Results and Discussion

### 3.1. Morphology

**Figure 2:**
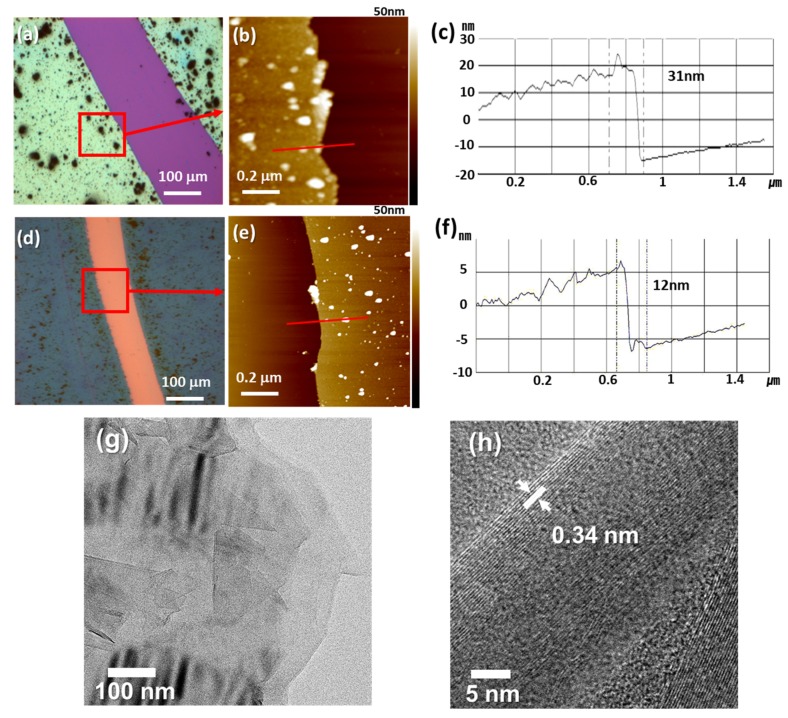
Optical images and tapping-mode atomic force microscopy (AFM) results of (**a**–**c**) polydopamine (PDA) and (**d**–**f**) C-PDA. (**g**,**h**) High-resolution TEM images of the C-PDA film.

The morphologies and structures of the as-prepared C-PDA were investigated by TEM and AFM. The AFM analyses (Figure 2a–c) confirm that the PDA forms a uniform coating on the Si substrate with a thickness of approximately 31 nm. After carbonization, the thickness of the C-PDA decreases to approximately 12 nm (Figure 2d–f). It is noteworthy that the white islands in the AFM image are consistent with the carbon aggregations on the sheet during the annealing process. Figure 2g shows the TEM images of the C-PDA sheet, which was heated at 800 °C for 1 h at the heating rate of 10 °C/min. It is interesting to note that there are several dark domains distributed in the C-PDA sheet, which consists of folding of a graphite-like thin film. Because of the layered structure of graphitic carbon, we can expect that the nanosized sheet not only functions as a supporting layer with high conductivity but also creates homogeneous vacancies inside the sheet planes. In the previous reports, the C-PDA structure usually exhibits micropores after the calcination steps [42,43,55], which would be beneficial to electrocatalytic mass transfer and gas diffusion. Moreover, ultrathin C-PDA nanosheets enable shorter pathways for charge migration from the bulk material to the reaction sites located on its surfaces. Therefore, charge carriers can travel faster in the material and on their surfaces to reach the active sites, thus enhancing the electrocatalytic efficiency. Additionally, we carefully measured the layer distance of these thin films and found that it is approximately 0.34 nm, which is the typical distance between graphite stacking layers (Figure 2h) [56]. 

### 3.2. Raman and XPS

**Figure 3:**
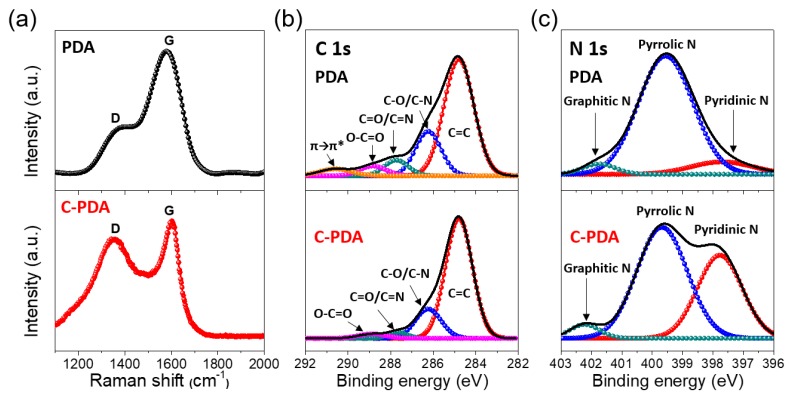
(**a**) Raman spectra and (**b,c**) X-ray photoelectron spectroscopy (XPS) survey of PDA and C-PDA prepared samples.

Raman spectroscopy was performed to study the structure and electronic properties of the C-PDA nanosheet (Figure 3a). The two peaks, one at approximately 1380 cm^−1^ and the other at 1590 cm^−1^, were assigned to the D (disordered carbon) and G bands (graphitic carbon), respectively [38,39,48]. It is worthwhile to note that the intensity ratio of the D and G bands, I_D_/I_G_, in the PDA film was 0.69, which is significantly low. However, after heat treatment during annealing, in comparison to the D peak (I_D_/I_G_ = 0.97), the G peak indicated a generation of defects and edges. In addition, this increase in the intensity ratio also indicates doping by heteroatoms (nitrogen and oxygen) in the graphitic planes during the calcination of PDA [38]. The chemical structural analysis of PDA and C-PDA was evaluated by the X-ray photoelectron spectroscopy (XPS) spectrum (Figure 3b,c). The peaks at approximately 284.5, 400.9, and 533.5 eV correspond to the C1s, N1s, and O1s core levels, respectively. The survey scan illustrates the existence of 6.2% N and 5.9% O in C-PDA, which are lower than the typical values in PDA (8.8% N and 14.9% O), thus indicating the efficiency of thermal reduction during annealing. For the C1s spectrum, the peaks at 284.7, 286.3, 287.8, 288.9, and 290.5 eV correspond to the C−C, C−O/ C−N, C=O/C=N, O−C=O, and π → π* transitions, respectively. The high-resolution N1s spectra can be deconvoluted into three peaks located at 397.8, 399.6, and 402.1 eV, which can be assigned to the pyridinic N, pyrrolic N, and graphitic N, respectively. Obviously, the N1s spectrum for PDA is solely dominated by pyrrolic nitrogen. Meanwhile, the XPS survey of C-PDA exhibited a significant peak area of pyridinic nitrogen, which is comparable with that of pyrrolic nitrogen, thus indicating the appearance of nitrogen at the edge (or defect) of the graphitic layer. This change indicates the partial formation of a disordered structure of graphene sheets by nitrogen doping. Some reports mention that the nitrogen in the defect of the N-doped carbon is beneficial for hydrogen generation due to its high electronegativity [57,58]. 

### 3.3. Electrochemical Performance

**Figure 4:**
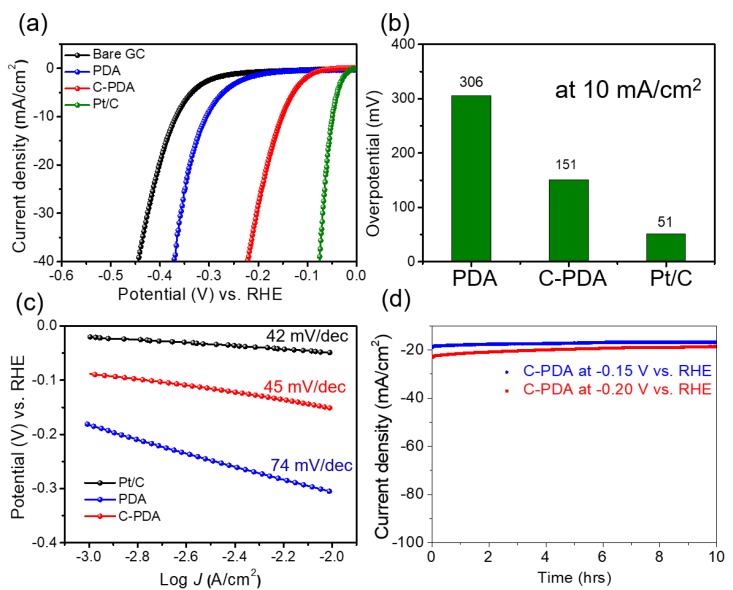
Electrochemical performance of prepared samples. (**a**) The hydrogen-evolution reaction (HER) polarization curves, (**b**) the overpotential value at 10 mA cm^−2^, (**c**) the corresponding Tafel slope of C-PDA samples, and (**d**) the chronoamperometric operation of C-PDA electrocatalyst at the potential of −0.15 V and −0.20 V vs. RHE during 10 hours, respectively.

The electrochemical HER activity of the prepared samples was investigated by depositing them on a GC with a catalyst loading of 0.13 mg cm^−2^. The experiment was conducted using 1 M perchloric acid electrolyte (pH approximately 0.3) in a three-electrode configuration. Figure 4a shows the linear sweep voltammetry (LSV) curves of different PDA catalysts after iR compensation. For the case of onset overpotential (defined here as the overpotential η at 1.0 mA cm^−2^), the pristine PDA exhibited a high value of 176 mV, which is close to the overpotential value of bare GC (203 mV). This value indicates that neither the GC nor the PDA is a satisfactory catalyst for the HER. In contrast, the onset overpotential of C-PDA was 68 mV, which is significantly lower than that of PDA, thus indicating that C-PDA is one of the best reported non-noble catalysts for use in acidic media to date. In addition, the −0.13 V operating potential of C-PDA, which is significantly lower than that of the PDA sample (−0.28 V), delivers a cathodic current density of 5 mA cm^−2^, thus confirming the unique role of N-doped carbon nanosheets in electrocatalytic processes. This potential value has largely outperformed those reported by previous literature for doped carbon-based materials (Table 1). It should be noted that compared to commercial Pt/C (5 wt.%, Alfa), which is the benchmark catalyst for the HER, the activity of C-PDA still underperforms (90 mV at 5 mA cm^−2^). At 10 mA cm^−2^, the overpotential of PDA and C-PDA are 306 and 151 mV, respectively (Figure 4b). Furthermore, C-PDA exhibits a low Tafel slope value of 45 mV decade^−1^ (Figure 4c), which is also smaller than that of PDA (74 mV decade^−1^) and somewhat comparable with that of Pt/C (42 mV decade^−1^), thus implying favorable HER kinetics by N-doping at the edge of the graphite layer. A small Tafel slope also suggests that the reaction proceeds through the Volmer–Heyrovsky mechanism, in which the electrochemical desorption step is rate-limiting. As discussed above, the significantly improved catalytic activity of the C-PDA film is the result of a potent combination of the following three factors: (i) the decrease in the thickness of the PDA film after calcination, which not only increases the specific surface area of material but also enables the rapid diffusion of charges/species during the reaction; (ii) the change in the chemical structure of the polymer (PDA) to a carbon network (C-PDA), which enables smaller charge-transfer resistances of the electrode; and (iii) the existence of defected nitrogen atoms that in turn contribute to the high abundance of available catalytically active sites. In addition, the long-term durability of the C-PDA electrocatalyst for HER was investigated at the potential of −0.15 V and −0.20 V vs. RHE (Figure 4d). The current density to time (*J-t*) curves maintained more than 80% of its initial current density value of approximately −20 mA cm^−2^ over 10 h.

### 3.4. Theoretical Calculation

**Figure 5:**
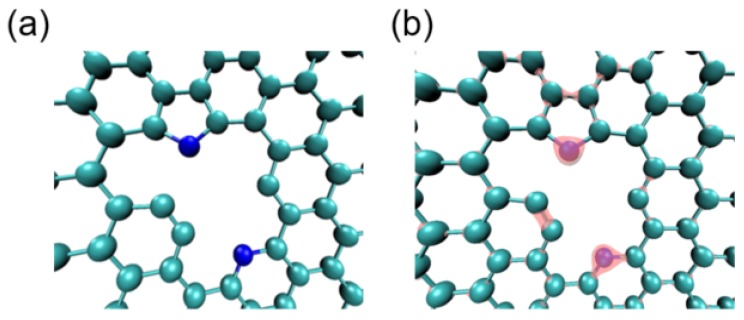
(**a**) Proposed model and (**b**) charge density distribution of N-doped carbon models. Isosurface value for the model is 5 × 10^−6^ e Å^−3^. For the spheres in the model, cyan indicates carbon, and the blue indicates nitrogen, while hydrogen is not shown in the model. Herein, we construct a nitrogen-doped carbon model based on the XPS results.

Given that N-doped carbon is a highly active catalyst for use in the HER, it is accepted that the N atoms in the carbon network are important for the water molecule-splitting process. To confirm the origin of the activity enhancement in this doped model, we performed DFT calculations to investigate the promotion effect of the primary N-doping. Hence, the graphene framework doped by N atoms at different sites was constructed according to the XPS characterizations (Figure 5a). It is well-known that doping carbon with heteroatoms of different electronegativities can adjust the electron donor-acceptor behavior and thus improve the apparent catalytic activities of the doped samples [66]. Owing to the clearly distinguishable electronegativities of N (3.04) and C (2.55), the charge transfer between N and C is significant. Interestingly, after doping, the partial charge of nitrogen is more negative, while those of the neighboring carbons are more positive (Table 2).

Additionally, our calculation results demonstrate that the charge density differences around nitrogen atoms at different sites are highly divergent. Specifically, the partial charge of –N= exhibits a value of −0.246 while the partial charge of –N-H exhibits a value of −0.275. These phenomena suggest that such a significant activity enhancement is caused by not only the N-doping but also the various sites of such N atoms. Thus, the catalytic enhancement can be attributed to the change in the electron charge density. Previous theoretical studies on graphene-based materials have confirmed that the adsorption strengths of H*, which are the key reaction intermediates of the HER, exhibit a correlation with the electron density difference [67,68]. Hence, a large number of carbon atoms could serve as electrocatalytically active sites to adsorb the H* reaction intermediates after tertiary/aromatic N-doping. Consequently, the apparent activity of the HER is remarkably boosted compared to that achieved with single doping.

## 4. Conclusions

In summary, due to the advantages of PDA chemistry, we have presented a novel strategy to produce N-doped nanocarbons as a potential candidate for the hydrogen-evolution reaction. C-PDA nanosheets exhibit outstanding electrocatalytic activity during the HER. The combination of experimental data and DFT calculations further confirms that such excellent activity of the N–doped carbon originates from the formation of a large number of electrocatalytically active sites. The required overpotential of the C-PDA at 5 mA/cm^2^ is 130 mV, and its Tafel slope is 45 mV/decade. Experimental characterizations confirm that the excellent performance of the nanosheets can be attributed to multisite nitrogen doping, while theoretical computations confirm the promotion effect of tertiary/aromatic nitrogen doping in enhancing the spin density of doped samples, and consequently in forming highly electroactive sites for HER application. Our approach in this study has resulted in a new design strategy to prepare non-noble catalysts exhibiting high efficiency for hydrogen production.

## Figures and Tables

**Table 1:** Comparison of the recent carbon-based materials for HER performance.

Catalyst	Substrate	η_onset_ (mV vs. RHE)	Tafel Slope (mV/dec)	Electrolyte	Ref.
N-carbon	GCE *	343	187	1M KOH	[41]
N-carbon	GCE	307	170	1M KOH	[58]
N-carbon	GCE	387	162	1M KOH	[59]
N-graphene	RDE **	543	143	0.1M KOH	[60]
N-carbon	GCE	165	228	0.5M H_2_SO_4_	[58]
N-carbon	GCE	487	159	0.5M H_2_SO_4_	[59]
N-carbon	RDE	30	43	0.5M H_2_SO_4_	[61]
N-carbon	Graphene Foam	300	147	0.5M H_2_SO_4_	[62]
N-graphene	RDE	378	116	0.5M H_2_SO_4_	[60]
P-graphene	RDE	452	133	0.5M H_2_SO_4_	[60]
P-graphene	RDE	601	159	0.1M KOH	[60]
N,S-carbon	GCE	259	133	1M KOH	[41]
N,S-carbon	GCE	201	77	0.5M H_2_SO_4_	[59]
N,S-carbon	GCE	292	103	1M KOH	[59]
N,S-carbon	GCE	179	121	0.5M H_2_SO_4_	[63]
N,S-carbon	GCE	312	180	0.5M H_2_SO_4_	[64]
N,P-carbon	GCE	442	139	0.5M H_2_SO_4_	[59]
N,P-carbon	GCE	352	106	0.5M H_2_SO_4_	[65]
N,P-graphene	RDE	320	91	0.5M H_2_SO_4_	[60]
N,P-graphene	RDE	497	145	0.1M KOH	[60]
N,P-carbon	GCE	418	118	1M KOH	[59]
N,B-carbon	GCE	523	198	0.5M H_2_SO_4_	[59]
N,B-carbon	GCE	601	152	1M KOH	[59]
N-carbon	GCE	68	45	1M HClO_4_	This work

* GCE: glassy carbon electrode, ** RDE: rotating disk glassy carbon electrode.

**Table 2:** Calculated parameters of proposed models.

Model	Partial Charge of N	Partial Charge of Neighbor C	Dipole Moment (Debye)
–NH_2_	−0.362	0.038	1.672
–N–H	−0.356	0.046 ± 0.008	1.915
–N=	−0.325	0.077 ± 0.032	0.762
